# TGF-β1 and its signal molecules: are they correlated with the elasticity characteristics of breast lesions?

**DOI:** 10.1186/s12885-021-09036-4

**Published:** 2021-12-15

**Authors:** Meng Ke Zhang, Bo Wang, Shi Yu Li, Gang Liu, Zhi Li Wang

**Affiliations:** 1 Department of Ultrasound, the First Medical Center, Chinese PLA General Hospital, 28 Fuxing Road, Beijing, 100853 China; 2 Department of Radiology, the First Medical Center, Chinese PLA General Hospital, 28 Fuxing Road, Beijing, 100853 China

**Keywords:** TGF-β1, Breast lesions, Elasticity parameters, Signal molecules

## Abstract

**Background:**

Shear wave elastography can evaluate tissue stiffness. Previous studies showed that the elasticity characteristics of breast lesions were related to the components of extracellular matrix which was regulated by transforming growth factor beta 1(TGF-β1) directly or indirectly. However, the correlation of the expression level of TGF-β1, its signal molecules and elasticity characteristics of breast lesions have rarely been reported. The purpose of this study was to investigate the correlation between the expression level of TGF-β1, its signal molecules, and the elasticity characteristics of breast lesions.

**Methods:**

135 breast lesions in 130 patients were included. Elasticity parameters, including elasticity modulus, the elasticity ratio, the “stiff rim sign”, were recorded before biopsy and surgical excision. The expression levels of TGF-β1 and its signal molecules, including Smad2/3, Erk1/2, p38 mitogen-activated protein kinase (MAPK), c-Jun N-terminal kinase 2 (JNK2), phosphoinositide 3-kinase (PI3K), and protein kinase B (PKB/AKT) were detected by immunohistochemistry. The diagnostic performance of the expression level of those molecules and their correlation with the elasticity characteristics were analyzed.

**Results:**

Elasticity parameters and the expression levels of TGF- β1 and its signal molecules of benign lesions were lower than those of malignant lesions (*P*<0.0001). The expression levels of TGF- β1 and its signal molecules were correlated with elasticity parameters. The expression levels of TGF- β1 and its signal molecules in lesions with “stiff rim sign” were higher than those without “stiff rim sign” (*P*<0.05). And the expression levels of Smad2/3, Erk1/2, p38 MAPK, JNK2, PI3K and AKT were correlated with that of TGF- β1. The area under the curve for receiver operator characteristic curve of TGF-β1 and its signal molecules in the differentiation of malignant and benign breast lesions ranged from 0.920–0.960.

**Conclusions:**

The expression levels of TGF-β1, its signal molecules of breast lesions showed good diagnostic performance and were correlated with the elasticity parameters. The expression levels of signal molecules were correlated with that of TGF- β1, which speculated that TGF- β1 might play an important role in the regulation of breast lesion elasticity parameters and multiple signal molecule expressions.

**Supplementary Information:**

The online version contains supplementary material available at 10.1186/s12885-021-09036-4.

## Introduction

Breast cancer is one of the most common cancer with the second-highest cancer-associated deaths among women worldwide [1, 2]. Studies showed that early detection of breast cancer in women improves prognosis in breast cancer survivors [3, 4]. At present, mammography is widely used as the main tool for breast screening. However, the sensitivity of mammography is relatively low in women with dense breast tissue, resulting in missed or delayed diagnosis [5]. Shear wave elastography (SWE), a newly ultrasound-based technology, can measure tissue stiffness and provides a qualitatively and quantitatively interpretable color-coded map [6]. Many studies indicated that SWE had good diagnostic accuracy in the differentiation of benign and malignant breast lesions [6–8].

Previous studies showed that the components of extracellular matrix (ECM) were related to the elasticity characteristics of breast lesions, and collagen and elastin in ECM were important factors that determine the elasticity of breast lesions [9, 10]. It is reported that the changes of composition and structure of ECM are mainly regulated by transforming growth factor β (TGF-β) [11, 12]. TGF-β, which is widely distributed in human body, has 3 isoforms, among which TGF- β1 is the most abundant. It can regulate the processes of cell carcinogenesis, proliferation, differentiation, apoptosis, metabolism and growth through TGF-β1/Smad, TGF-β1/ mitogen-activated protein kinase (MAPK), phosphoinositide 3-kinase (PI3K)/ protein kinase B (PKB/AKT) and other signal transduction pathways, and participate in almost the whole process of occurrence, development, invasion and metastasis of breast lesions [13–15]. In addition, TGF-β1 can directly or indirectly promote the excessive deposition of collagen and fibrin in ECM, inhibit the degradation of ECM, and increase ECM stiffness [16–18]. Therefore, TGF-β1, ECM, and elasticity characteristics of breast lesions are closely related.

However, it is rarely reported whether there is a correlation between TGF-β1, its signal molecules, and the elasticity parameters of breast lesions. Therefore, the purpose of this study was to investigate the relationship of the expression levels of TGF-β1, its signal molecules, and elasticity parameters of breast lesions.

## Materials and methods

### Patients

This study was conducted in accordance with the Declaration of Helsinki (as revised in 2013). The study was approved by the medical ethics committee of our hospital (No. S2020–336-01), and written informed consent was obtained from all patients.

135 breast lesions in 130 patients who underwent ultrasound-guided vacuum-assisted biopsy (VAB) or core needle biopsy (CNB) or surgical excision (mastectomy, breast-conserving surgery) after SWE examinations were included in this study from March 2018 to October 2018. The pathological result was considered as the “gold standard”. Then the axillary lymph node metastasis of patients with malignant breast lesions was followed up.

The patients were included if they met the following criteria: I. Pathological results were obtained by VAB or surgical excision; II. Patients haven’t undergone neoadjuvant chemotherapy or radiotherapy; III. Patients did not have other malignant lesions or serious diseases of the heart, lung, liver, kidney, etc. IV. Patients had comprehensive information of clinical, ultrasound, pathology prognosis and follow-up;

### SWE examination

Aixplorer ultrasound system (SuperSonic Imagine, Aix en Provence, France) with an L15–4 linear array probe (4.0–15.0 MHz) was used for 2D-SWE examination (scale 0-300Kpa). The SWE examination was performed by an experienced radiologist (Z.L.W) with more than 15 years’ working experience in breast ultrasound. Breast lesions were located by conventional ultrasound and placed in the center of the screen. During SWE scan, the probe was positioned perpendicular, and the probe was maintained to a minimum pressure. The patients were asked to breathe gently during the examination in order to minimize the motion artifact. The image was acquired if it was stabilized for 3 s. To measure the accurate stiffness of the lesion, an appropriate region of interest (ROI) was chosen to cover all parts of the lesion, including the stiffest part outside the lesion. Then the maximum elasticity modulus (Emax), mean elasticity modulus (Emean), minimum elasticity modulus (Emin), the standard deviation of elasticity modulus (Esd) was recorded. The elasticity ratio (Eratio) of the lesion and the surrounding normal breast tissue at the same depth was also recorded. The examination was repeated in five different sections of the lesion and the mean value of Emax, Emean, Emin, Esd and Eratio was recorded. The elastography parameter of “stiff rim sign”, defined as the red area of increased stiffness with or without an open or a closed ring at the edge of the lesion, was also recorded.

### Immunohistochemistry

The samples were fixed in formalin and embedded in paraffin, and then cut into sections with a thickness of 4 μm, and then TGF-β1, Smad2/3, Erk1/2, p38 MAPK, JNK2, PI3K and AKT expression was evaluated by immunohistochemistry. Image-Pro Plus 6.0 was used for semi-quantitative analysis of immunohistochemical results. Five ROIs were randomly selected from each sample under the 400× field of view and photographed to measure the integrated optical density (IOD) and area. The yellow area is the positive expression area. Expression levels of TGF- β 1 and its signal molecules were expressed by average optical density (IOD/area).

### Statistical analysis

SPSS 26.0, standard version (SPSS Inc., Chicago, IL, USA) statistical software was used for statistical analysis. The quantitative data were expressed as (mean ± standard deviation, x ± s) and the qualitative data were expressed as percentage. The Student’s t-test was used to compare the differences between groups of quantitative data, and χ^2^ test was used to compare the differences of qualitative data. Taking the pathological results as the “gold standard”, the receiver operator characteristic curve (ROC) of each factor were drawn respectively, and the efficacy was evaluated by the area under the curve (AUC), and the cutoff value, sensitivity, and specificity were analyzed, and the differences among AUC were compared by Z test. Spearman rank correlation test was used for correlation analysis. Quantile regression was used to estimate the associations between the TGF- β 1, its signal molecules and elasticity modules at the 0.05–0.95 quantiles. Logistic regression analysis was used to estimate the associations between the TGF- β 1, its signal molecules and the “stiff rim sign”. *P*<0.05 was considered as the difference was statistically significant.

## Results

### Study population

The age of the patients ranged from 18 to 73 years, with an average of (44 ± 12) years, and the maximum diameter of the lesion ranged from 0.5 to 3.9 cm, with an average of 2.1cm. Of the 135 breast lesions, 84 (62.2%, 84/135) were benign, including 32 fibroadenomas, 40 adenoses, 3 intraductal papillomas, 8 inflammatory lesions, 1 benign phyllodes tumor, and 51 (37.8%, 51/135) were malignant, including 46 invasive carcinomas, 3 intraductal carcinomas and 2 mucinous carcinomas.

### Analysis of elasticity parameters of breast lesions

**Fig. 1:**
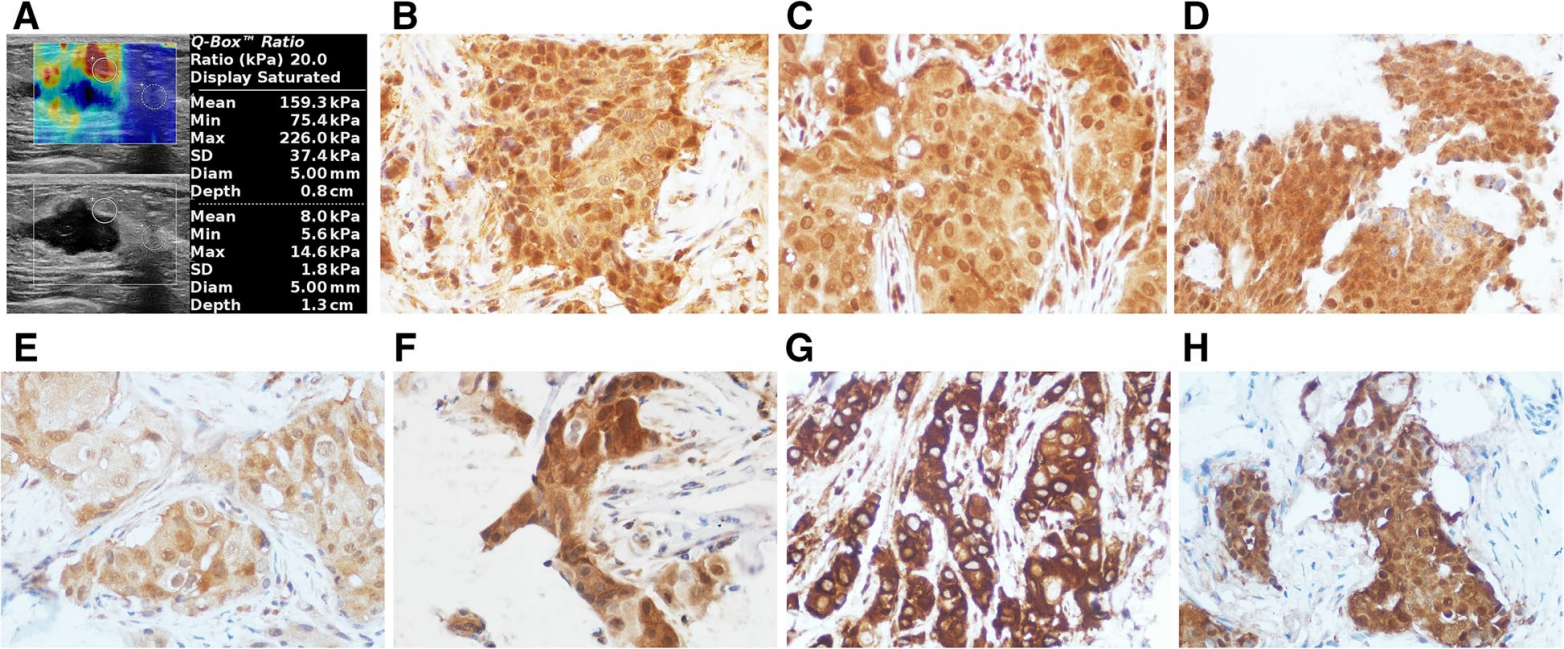
SWE and immunohistochemical images of invasive breast cancer in a 38-year-old woman. **A**: SWE showed that the Emax was 226.0 kPa, the Emean was 159.3 kPa, the Esd was 37.4 kPa, and the Eratio was 20.0, and the “stiff rim sign” can be seen; **B** ~ **H**: Immunohistochemical staining showed that the expression of TGF- β1, Smad2/3, Erk1/2, p38 MAPK, JNK2, PI3K and AKT was strong or moderate positive, and the average optical density was 0.349, 0.342, 0.355, 0.172, 0.296, 0.373, 0.324, respectively (× 400)

**Fig. 2:**
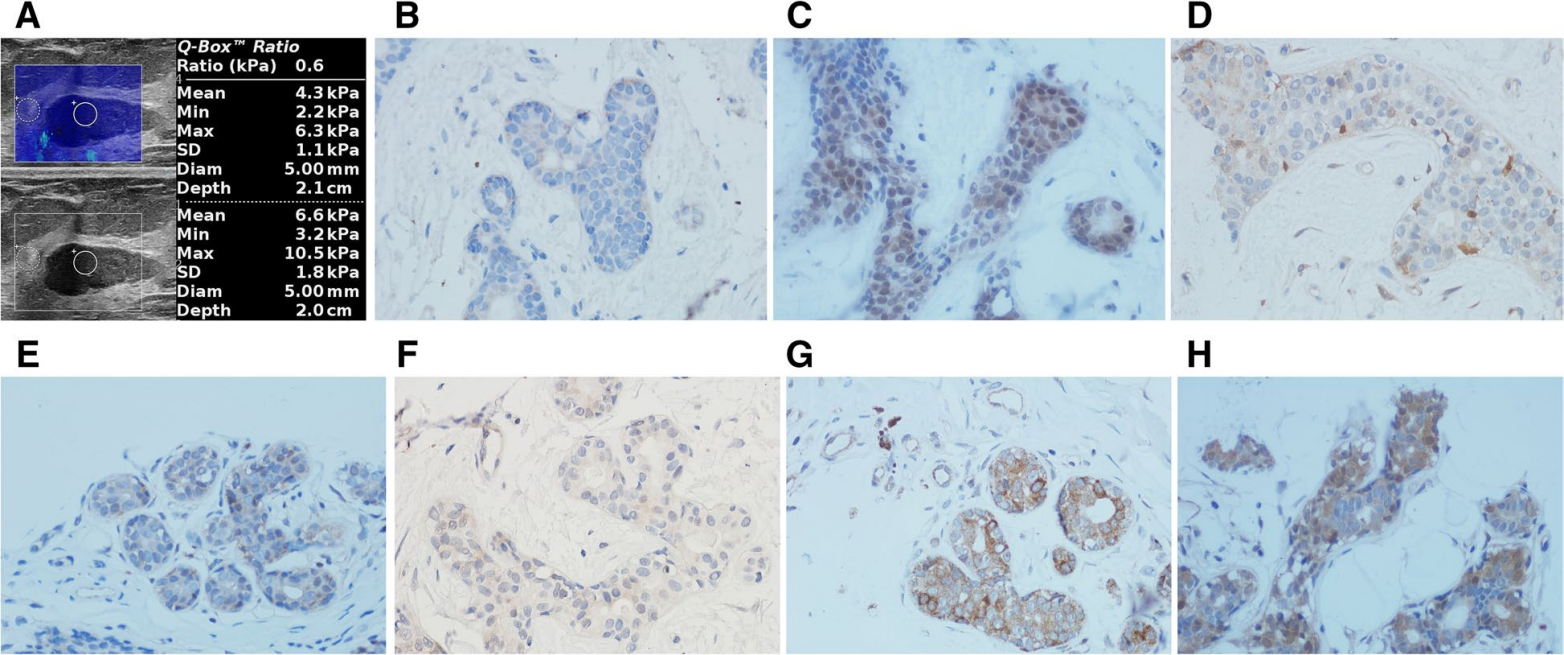
SWE and immunohistochemical images of breast fibroadenoma in a 36-year-old woman. **A**: SWE showed that the Emax was 6.3 kPa, the Emean was 4.3 kPa, the Esd was 1.1 kPa, and the Eratio was 0.6; **B** ~ **H**: Immunohistochemical staining showed that the expression of TGF- β1, Smad2/3, Erk1/2, p38 MAPK, JNK2, PI3K and AKT was weak positive, and the average optical density was 0.012, 0.078, 0.032, 0.022, 0.050, 0.090, 0.099, respectively (× 400)

The elasticity characteristics of benign and malignant breast lesions were shown in Table 1, Fig. 1A, Fig. 2A, and Supplementary material 1. The Emax, Emean, Esd and Eratio of benign breast lesions were significantly lower than those of malignant lesions (*P* < 0.001), but there was no significant difference in Emin between benign and malignant breast lesions (*P* = 0.202). Besides, the detection rate of “stiff rim sign” in malignant lesions was significantly higher than that of benign lesions (*P* < 0.001).

**Table 1:** Comparison of elastic characteristics between benign and malignant breast lesions

Factors	Benign lesions (*n* = 84)	Malignant lesions (*n* = 51)	t (t’)/χ^2^	*P*
**Emax (kPa)**^a^	58.2 ± 50.5	161.9 ± 79.5	9.276	<0.001
**Emean (kPa)**^a^	35.9 ± 26.4	99.6 ± 51.9	9.433	<0.001
**Emin (kPa)**	15.4 ± 12.9	20.6 ± 19.5	1.287	0.202
**Esd (kPa)**^a^	8.9 ± 7.9	30.1 ± 17.5	9.011	<0.001
**Eratio**^a^	2.1 ± 1.7	6.0 ± 4.1	7.625	<0.001
**Stiff rim sign [***n***(%)]**^a^	4 (4.8)	38 (74.5)	72.030	<0.001

^a^statistical significance

### Expression levels of TGF- β 1 and its signal molecules in breast lesions

The expression levels of TGF- β1 and its signal molecules in breast lesions were presented in Table 2, Fig. 1B~H and Fig. 2B~H. Immunohistochemical staining showed that TGF- β1 and PI3K were mainly expressed in the cytoplasm, while Smad2/3, Erk1/2, p38 MAPK, JNK2 and AKT were mainly expressed in both cytoplasm and nucleus. And the expression levels of TGF- β1 and its signal molecules in malignant breast lesions were significantly higher than those in benign lesions (*P* < 0.001).

**Table 2:** Comparison of expression levels of TGF-β1 and other factors between benign and malignant breast lesions (x ± s)

Factors	Benign lesions (*n* = 84)	Malignant lesions (*n* = 51)	t (t’)	*P*
**TGF-β1**^a^	0.1038 ± 0.0092	0.2995 ± 0.01114	13.300	<0.001
**Smad2/3**^a^	0.0745 ± 0.0497	0.2511 ± 0.0754	16.160	<0.001
**Erk1/2**^a^	0.1242 ± 0.0552	0.2547 ± 0.0667	12.300	<0.001
**p38 MAPK**^a^	0.0459 ± 0.0516	0.2613 ± 0.0914	15.930	<0.001
**JNK2**^a^	0.1101 ± 0.0549	0.2558 ± 0.0720	12.450	<0.001
**PI3K**^a^	0.1240 ± 0.0702	0.3425 ± 0.0608	16.640	<0.001
**AKT**^a^	0.0741 ± 0.0546	0.2166 ± 0.0679	13.070	<0.001

^a^statistical significance

**Fig. 3:**
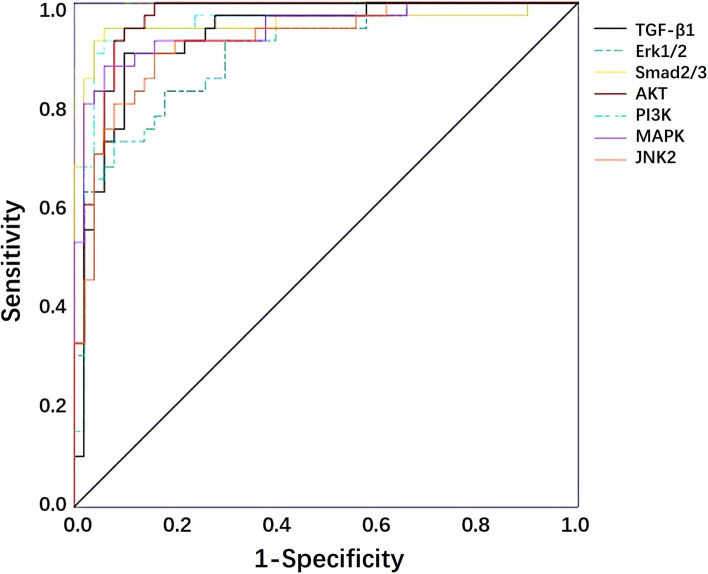
ROC curves of TGF-β1, Smad2/3, Erk1/2, p38 MAPK, JNK2, PI3K and AKT expression levels in breast lesions for differential diagnosis of benign and malignant breast lesions

The AUC of the expression levels of TGF-β1 and Smad2/3, Erk1/2, p38 MAPK, JNK2, PI3K and AKT for the differential diagnosis of benign and malignant breast lesions were 0.931 (0.874–0.967), 0.953 (0.901–0.982), 0.920 (0.861–0.960), 0.957 (0.902–0.986), 0.934 (0.874–0.971), 0.960 (0.901–0.989) and 0.939 (0.883–0.974), respectively (Fig. 3). Z test showed that there was no significant difference among those groups (*P* > 0.05). The cutoff value, sensitivity and specificity of TGF- β1 and its signal molecules for the differentiation of benign and malignant breast lesions were shown in Supplementary material 2.

### Correlation analysis

The correlation of the expression levels of TGF-β1, its signal molecules and elasticity characteristics in breast lesions was shown in Supplementary material 3. Spearman test showed that the expression levels of TGF-β1 and Smad2/3, Erk1/2, p38 MAPK, JNK2, PI3K and AKT were positively correlated with Emax, Emean, Esd and Eratio in breast lesions (correlation coefficient = 0.879, 0.595, 0.571, 0.562, 0.516, 0.552, 0.619 for Emax, 0.841, 0.549, 0.503, 0.504, 0.516, 0.542, 0.578 for Emean, 0.865, 0.580, 0.566, 0.593, 0.514, 0.559, 0.649 for Esd and 0.746, 0.510, 0.507, 0.506, 0.536, 0.511, 0.550 for Eratio, *P*<0.0001).

Expression levels of TGF-β1 and its signal molecules in breast lesions with or without the “stiff rim sign” were shown in Table 3. Immunohistochemical staining showed that the expression levels of TGF-β1 and its signal molecules in breast lesions with “stiff rim sign” were significantly higher than those without “stiff rim sign” (*P*<0.001).

**Table 3:** Comparison of expression levels of TGF-β1 and other factors in breast lesions with and without “stiff rim sign” (x ± s)

Factors	Stiff rim sign		t (t’)	*P*
	Yes(***n*** = 42)	None(***n*** = 93)		
**TGF-β1**^a^	0.3090 ± 0.0682	0.1164 ± 0.0960	11.720	<0.001
**Smad2/3**^a^	0.2332 ± 0.0981	0.1004 ± 0.0792	8.242	<0.001
**Erk1/2**^a^	0.2441 ± 0.0831	0.1416 ± 0.0683	7.539	<0.001
**p38 MAPK**^a^	0.2429 ± 0.1116	0.0832 ± 0.0998	7.673	<0.001
**JNK2**^a^	0.2429 ± 0.0875	0.1285 ± 0.0729	7.321	<0.001
**PI3K**^a^	0.3212 ± 0.0954	0.1692 ± 0.1096	7.130	<0.001
**AKT**^a^	0.2025 ± 0.0886	0.0945 ± 0.0712	7.332	<0.001

^a^statistical significance

The correlation between the expression levels of TGF-β1 and its signal molecules in breast lesions was shown in Supplementary material 3. Spearman test showed that the expression levels of Smad2/3, Erk1/2, p38 MAPK, JNK2, PI3K and AKT were positively correlated with TGF-β1 in breast lesions (correlation coefficient = 0.678, 0.633, 0.645, 0.611, 0.589, 0.663, *P*<0.0001).

### Prediction of axillary lymph node metastasis

Expression levels of TGF-β1 and its signal molecules in malignant breast lesions with or without axillary lymph node metastasis were presented in Table 4. The expression levels of all factors were significantly higher in malignant breast lesions with axillary lymph node metastasis than those without axillary lymph nide metastasis (*P* < 0.05).

**Table 4:** Comparison of expression levels of TGF-β1 and other factors in malignant breast lesions with and without axillary lymph node metastasis (x ± s)

Factors	Metastasis		t (t’)	*P*
	Yes(***n*** = 15)	None(***n*** = 36)		
**TGF-β1**^a^	0.3204 ± 0.0381	0.2648 ± 0.0650	2.999	0.0048
**Smad2/3**^a^	0.2956 ± 0.0251	0.2513 ± 0.0628	2.592	0.0136
**Erk1/2**^a^	0.2814 ± 0.0609	0.2414 ± 0.0553	2.118	0.0410
**p38 MAPK**^a^	0.2999 ± 0.0506	0.2484 ± 0.0759	2.180	0.0363
**JNK2**^a^	0.2832 ± 0.0627	0.2415 ± 0.0507	2.063	0.0478
**PI3K**^a^	0.3396 ± 0.0316	0.3110 ± 0.0459	2.094	0.0436
**AKT**^a^	0.2225 ± 0.0716	0.1704 ± 0.0713	2.154	0.0382

^a^statistical significance

**Fig. 4:**
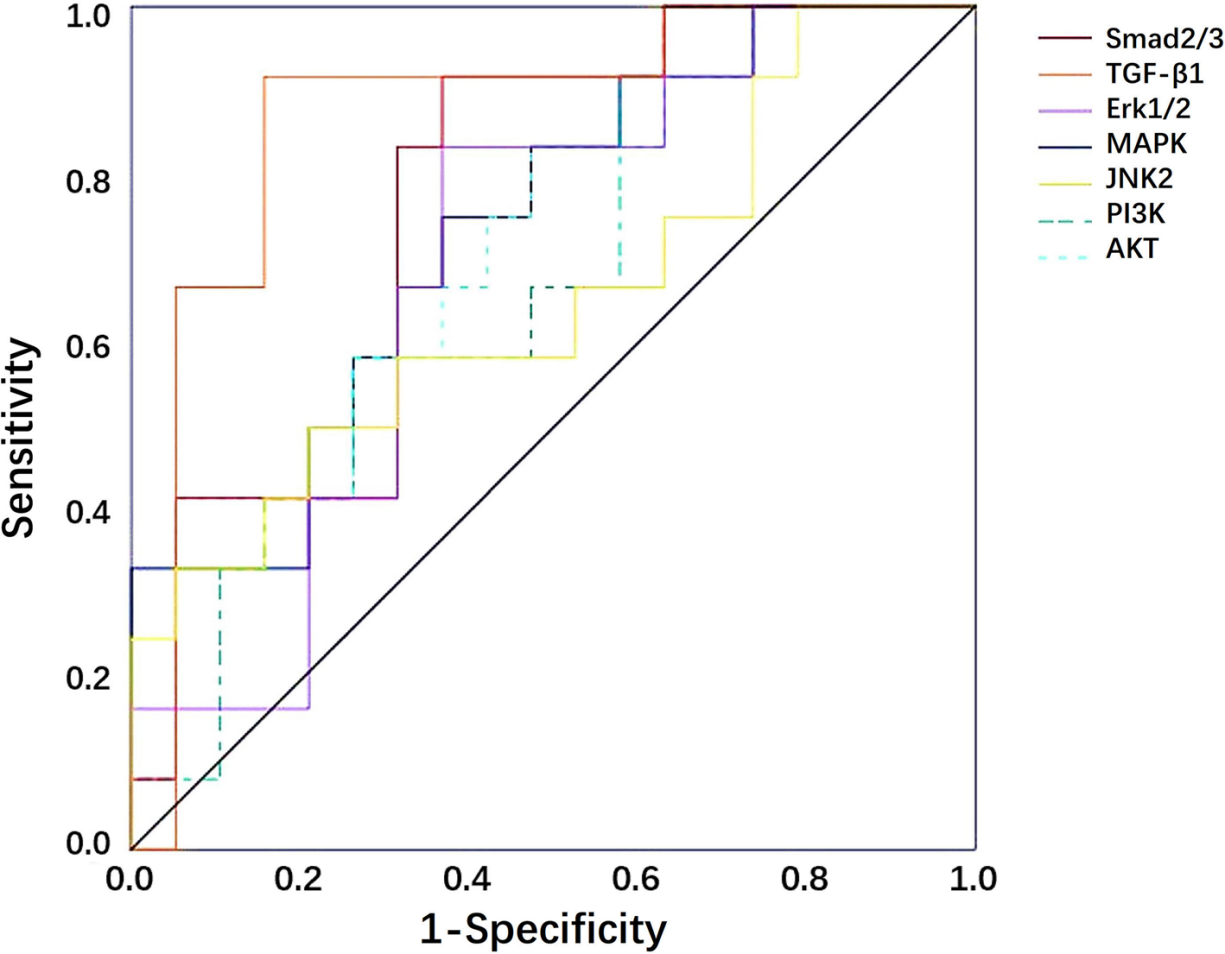
ROC curves of TGF-β1, Smad2/3, Erk1/2, p38 MAPK, JNK2, PI3K and AKT expression levels in malignant breast lesions for differential diagnosis of malignant breast lesions with and without axillary lymph node metastasis

Based on the expression levels of TGF- β1 and its signal molecules in malignant breast lesions, ROC curves for the prediction of axillary lymph node metastasis were shown in Fig. 4. The AUC for TGF-β1, Smad2/3, Erk1/2, p38 MAPK, JNK2, PI3K and AKT were 0.853 (0.703–0.946), 0.697 (0.529–0.834), 0.694 (0.527–0.832), 0.706 (0.531–0.845), 0.654 (0.466–0.813), 0.667 (0.493–0.813) and 0.689 (0.516–0.831), respectively. The cutoff value, sensitivity and specificity of TGF- β1 and its signal molecules for the prediction of axillary lymph node metastasis were shown in Supplementary material 4.

### Quantile regression analysis

As shown in Tables 5, 6, 7 and 8, the quantile regression analysis was performed with the expression levels of TGF- β 1and its signal molecules in breast lesions as independent variables and Emax, Emean, Esd and Eratio as target variables respectively. At different quantiles, only the expression level of TGF-β1 always had a significant positive effect on Emax, Emean, Esd and Eratio, while Smad2/3 only had a certain effect on Emean at the point of 0.75th quartile, a negative effect on Eratio at the point of 0.45th quartile. Erk1/2 only had a certain effect on Emean at the 0.75th quartile. Thus, the expression level of TGF-β1 is the most important factor to determine the Emax, Emean, Esd and Eratio of breast lesions.

**Table 5:** Quantile regression analysis results of Emax and the expression levels of TGF- β 1, Smad2/3, Erk1/2, p38 MAPK, JNK2, PI3K and AKT

Factors	Percentiles									
	0.05	0.15	0.25	0.35	0.45	0.55	0.65	0.75	0.85	0.95
Constant	0.879 (0.065)	9.246 (0.787)	5.892 (0.725)	2.208 (0.219)	5.293 (0.520)	8.165 (0.954)	4.873 (0.549)	7.216 (0.773)	6.928 (0.761)	12.269 (0.970)
TGF-β1	246.281 (4.088**)	394.203 (7.519**)	481.854 (13.296**)	508.789 (11.315**)	575.917 (12.679**)	593.743 (15.561**)	629.329 (15.912**)	637.585 (15.305**)	624.232 (15.366**)	838.401 (14.859**)
Erk1/2	120.037 (1.417)	17.122 (0.232)	37.736 (0.740)	35.942 (0.568)	5.311 (0.083)	8.878 (0.165)	7.135 (0.128)	−19.103 (−0.326)	10.072 (0.176)	−28.314 (− 0.357)
Smad2/3	−74.443 (1.113)	−16.223 (− 0.223)	−66.575 (− 1.322)	2.282 (0.037)	35.236 (0.558)	22.914 (0.432)	56.750 (1.033)	98.069 (1.940)	92.868 (1.859)	9.671 (0.123)
AKT	76.114 (1.115)	23.651 (0.364)	−4.522 (− 0.101)	−37.059 (− 0.665)	−33.669 (− 0.598)	−47.726 (− 1.009)	− 45.340 (− 0.924)	−46.166 (− 0.893)	1.183 (0.023)	6.457 (0.092)
PI3K	−59.305 (− 0.800)	−61.242 (− 0.950)	−57.044 (− 1.280)	−26.049 (− 0.471)	−34.215 (− 0.613)	−11.215 (− 0.239)	16.930 (0.348)	9.701 (0.189)	−4.563 (− 0.091)	23.487 (0.338)
p38 MAPK	−69.315 (− 1.103)	4.674 (0.086)	− 17.941 (− 0.475)	−20.672 (− 0.441)	2.370 (0.050)	37.668 (0.947)	45.589 (1.106)	22.639 (0.521)	−44.900 (− 1.060)	− 2.834 (− 0.048)
JNK2	34.819 (0.475)	−3.160 (− 0.050)	19.572 (0.444)	18.803 (0.344)	− 20.441 (− 0.370)	−72.192 (− 1.556)	−65.744 (− 1.299)	−94.286 (− 1.861)	− 88.809 (− 1.850)	−130.496 (− 1.902)

The values in parentheses were t value, *P<0.05, **P<0.01

**Table 6:** Quantile regression analysis results of Emean and the expression levels of TGF- β 1, Smad2/3, Erk1/2, p38 MAPK, JNK2, PI3K and AKT

Factors	Percentiles									
	0.05	0.15	0.25	0.35	0.45	0.55	0.65	0.75	0.85	0.95
Constant	2.515 (2.764**)	5.153 (0.614)	5.302 (0.607)	3.906 (0.535)	5.219 (0.937)	7.044 (1.129)	8.812 (1.174)	12.887 (1.893)	19.159 (2.447*)	52.857 (3.331**)
TGF-β1	165.740 (40.847**)	272.166 (7.273**)	275.848 (7.078**)	306.187 (9.404**)	311.012 (12.523**)	330.379 (11.878**)	331.917 (9.913**)	348.870 (11.493**)	396.475 (11.353**)	283.164 (4.001**)
Erk1/2	9.134 (1.869)	27.242 (0.518)	5.337 (0.097)	−19.645 (− 0.429)	−14.899 (− 0.427)	−26.436 (− 0.676)	− 62.152 (− 1.320)	−89.939 (− 2.107*)	−70.397 (− 1.434)	− 137.854 (− 1.385)
Smad2/3	−10.401 (− 1.948)	− 29.460 (− 0.590)	28.310 (0.523)	59.515 (1.316)	48.011 (1.391)	74.255 (1.921)	34.924 (0.900)	149.744 (3.550**)	87.728 (1.808)	167.575 (1.738)
AKT	6.943 (1.198)	58.972 (1.271)	−13.985 (− 0.289)	− 40.210 (− 0.996)	4.966 (0.161)	13.972 (0.405)	−5.790 (− 1.139)	− 41.723 (− 1.108)	− 20.310 (− 0.469)	−117.021 (− 1.333)
PI3K	− 9.712 (− 1.911)	− 15.839 (− 0.344)	− 26.241 (− 0.548)	2.051 (0.051)	−16.296 (− 0.534)	−22.552 (− 0.659)	−35.817 (− 0.870)	− 50.265 (− 1.347)	−63.175 (− 1.471)	− 176.770 (− 1.761)
P38 MAPK	−8.844 (− 1.818)	− 2.021 (− 0.052)	10.735 (0.264)	− 18.712 (− 0.551)	−6.704 (− 0.259)	−6.008 (− 0.207)	9.940 (0.285)	43.607 (1.378)	85.537 (2.349)	191.916 (1.836)
JNK2	9.514 (1.901)	−30.758 (− 0.676)	− 15.982 (− 0.337)	1.976 (0.050)	− 9.354 (− 0.310)	−22.872 (− 0.676)	8.012 (0.197)	33.942 (0.920)	−13.712 (− 0.323)	28.135 (0.327)

The values in parentheses were t value, *P<0.05, **P<0.01

**Table 7:** Quantile regression analysis results of Esd and the expression levels of TGF- β 1, Smad2/3, Erk1/2, p38 MAPK, JNK2, PI3K and AKT

Factors	Percentiles									
	0.05	0.15	0.25	0.35	0.45	0.55	0.65	0.75	0.85	0.95
Constant	0.527 (0.474)	−0.714 (− 0.311)	0.367 (0.156)	− 0.120 (− 0.049)	− 2.526 (− 0.757)	−2.043 (− 0.624)	−1.405 (− 0.423)	−2.446 (− 0.703)	−2.194 (− 0.503)	−2.805 (− 0.042)
TGF-β1	22.480 (4.946**)	70.439 (7.503**)	63.092 (6.578**)	75.920 (7.501**)	109.328 (8.017**)	113.801 (8.509**)	112.599 (8.286**)	104.303 (7.337**)	132.677 (7.440**)	117.040 (2.217*)
Erk1/2	12.786 (1.758)	−12.731 (− 0.891)	−9.527 (− 0.653)	−10.331 (− 0.671)	−2.835 (− 0.137)	− 10.140 (− 0.498)	−6.910 (− 0.334)	−4.137 (− 0.191)	5.001 (0.184)	18.186 (0.407)
Smad2/3	−10.225 (− 1.492)	− 25.332 (− 1.886)	−4.195 (− 0.306)	0.339 (0.023)	1.619 (0.083)	7.046 (0.368)	19.713 (1.014)	31.167 (1.532)	12.837 (0.503)	81.503 (1.912)
AKT	11.152 (1.653)	−3.635 (− 0.322)	2.482 (0.215)	1.541 (0.127)	−3.895 (− 0.237)	−7.848 (− 0.488)	− 11.279 (− 0.690)	13.315 (0.779)	2.485 (0.116)	−2.034 (− 0.37)
PI3K	−6.335 (− 1.163)	6.578 (0.585)	− 0.590 (− 0.051)	3.185 (0.263)	10.962 (0.671)	17.097 (1.067)	16.826 (1.033)	19.973 (1.172)	14.122 (0.661)	13.965 (0.319)
P38 MAPK	5.410 (1.138)	13.940 (1.374)	10.201 (0.984)	11.205 (1.024)	10.597 (0.719)	11.347 (0.785)	12.809 (0.872)	1.537 (0.100)	−18.073 (− 0.938)	−49.386 (− 1.073)
JNK2	− 11.226 (1.490)	7.915 (0.673)	5.724 (0.477)	−2.051 (− 0.162)	− 12.575 (− 0.736)	−15.636 (− 0.934)	−24.628 (− 1.447)	−18.915 (− 1.062)	9.965 (0.446)	22.565 (0.472)

The values in parentheses were t value, *P<0.05, **P<0.01

**Table 8:** Quantile regression analysis results of Eratio and the expression levels of TGF- β 1, Smad2/3, Erk1/2, p38 MAPK, JNK2, PI3K and AKT

Factors	Percentiles									
	0.05	0.15	0.25	0.35	0.45	0.55	0.65	0.75	0.85	0.95
Constant	− 0.058 (− 0.079)	−0.586 (− 0.946)	0.045 (0.077)	0.087 (0.182)	0.319 (0.687)	0.695 (0.980)	0.226 (0.284)	0.080 (0.136)	−0.119 (− 0.035)	4.236 (0.441)
TGF-β1	13.933 (4.601**)	6.751 (2.509*)	11.378 (4.419**)	14.082 (6.820**)	15.654 (7.768**)	14.928 (4.840**)	15.123 (4.366**)	18.126 (7.125**)	19.985 (7.339**)	110.466 (7.731**)
Erk1/2	2.771 (0.608)	6.286 (1.648)	0.249 (0.068)	−0.499 (−0.170)	−3.591 (−1.257)	− 3.766 (− 0.861)	−3.841 (− 0.782)	−0.037 (− 0.01)	0.913 (0.043)	−39.144 (− 0.662)
Smad2/3	−6.359 (− 1.417)	− 5.757 (− 1.533)	−4.420 (− 1.230)	−3.594 (− 1.247)	−6.242 (− 2.219*)	−6.249 (− 1.451)	− 2.435 (− 0.504)	−4.113 (− 1.158)	− 5.014 (− 0.241)	44.481 (0.764)
AKT	5.978 (1.500)	3.844 (1.152)	1.196 (0.375)	0.376 (0.147)	2.971 (1.189)	3.361 (0.878)	3.160 (0.736)	2.215 (0.702)	3.831 (0.207)	−15.238 (− 0.295)
PI3K	− 1.496 (− 0.378)	− 0.647 (− 1.196)	2.862 (0.904)	3.808 (1.500)	3.338 (1.234)	3.905 (1.209)	5.633 (1.322)	5.043 (1.612)	4.127 (0.225)	−13.009 (− 0.254)
P38 MAPK	− 2.112 (− 0.630)	0.385 (0.137)	−1.337 (− 0.498)	−1.521 (− 0.706)	− 2.104 (− 1.001)	− 0.774 (− 0.241)	−2.010 (− 0.557)	−0.933 (− 0.352)	−2.056 (− 0.132)	5.076 (0.117)
JNK2	5.940 (1503)	2.384 (0.721)	1.748 (0.552)	0.377 (0.149)	1.763 (0.712)	2.336 (0.616)	4.779 (1.122)	3.031 (0.969)	5.463 (0.298)	26.230 (0.511)

The values in parentheses were t value, *P<0.05, **P<0.01

### Logistic regression analysis

Taking the expression levels of TGF-β1, Smad2/3, Erk1/2, p38MAPK, JNK2, PI3K and AKT in breast lesions as independent variables and “stiff rim sign” as a dependent variable, multi-variable logistic regression analysis was performed. The logistic regression showed the expression level of TGF-β1 was the main factor determining the presence or absence of “stiff rim sign” (Table 9).

**Table 9:** The results of logistic regression analysis for determining the presence or absence of “stiff rim sign”

Factors	B	S.E.	Wald	***P***	OR	OR 95%CI
TGF-β1^a^	17.049	5.005	11.602	0.001	25,372,088.366	1392.462–462,305,423,321.366
Erk1/2	−3.985	7.253	0.302	0.583	0.019	0.000–27,728.468
Smad2/3	−5.140	6.000	0.734	0.392	0.006	0.000–750.258
AKT	6.854	5.871	1.363	0.243	947.442	0.010–94,159,513.24
PI3K	6.766	5.802	1.360	0.244	867.749	0.010–75,402,659.43
p38 MAPK	−1.128	4.253	0.070	0.791	0.324	0.000–1348.379
JNK2	2.039	5.253	0.151	0.698	7.686	0.000–227,464.510
Constant ^a^	−5.977	1.501	15.851	0.000	0.003	–

^a^statistical significance

## Discussion

SWE could quantitatively evaluate the elasticity characteristics of breast lesions and more accurately judge the benign and malignant breast lesions. Our study showed that there were significant differences in elasticity characteristics between benign and malignant breast lesions, which were the same as those of previous studies [7, 19]. In malignant breast lesions, tumor cells infiltrated into the surrounding tissue, causing tissue hyperplasia and fibrosis, which could lead to the accumulation of ECM components, rearrangement and cross-linking of ECM structure, and increased the stiffness of the lesions [20, 21].

Our study found that the expression levels of TGF-β1 and its signal molecules in malignant breast lesions were significantly higher than those in benign breast lesions. It was reported that TGF-β had both inhibitory and promoting effects on tumor cells [22]. In the early stage of tumorigenesis, TGF-β could induce tumor cell apoptosis and inhibit tumor growth through the TGF-β/Smad signal pathway. But the level of TGF-β was elevated by the secretion of most cancer cells in the late stage, and thus promote the occurrence and development of tumor [23]. The proliferation and invasion of cancer cells led to the activation of TGF-β1 and the increase of TGF-β1 expression, which affected the growth of cancer cells and promoted the transformation of normal fibroblasts into cancer associated fibroblasts (CAFs) [24, 25]. CAFs could interact with cancer ECM to promote the growth, development, invasion and metastasis of cancer, which further increased the expression level of TGF-β1. TGF-β1 participate in almost the whole process of occurrence, development, invasion and metastasis of breast lesions through TGF-β1/Smad, TGF-β1/MAPK, PI3K/AKT and other signal transduction pathways. Thus, the expression levels of TGF-β1 and its signal molecules in malignant breast lesions were higher than those in benign lesions. These also explained why the expression levels of TGF-β1 and its signal molecules in malignant breast lesions with or without axillary lymph node metastasis were significantly different.

This study also showed that the expression levels of TGF-β1 and its signal molecules in breast lesions had a certain value in the differential diagnosis of benign and malignant breast lesions, suggesting that TGF-β1 and its signal molecules might be used as new indexes for differential diagnosis of benign and malignant breast lesions and breakthrough points for clinical diagnosis and treatment.

In our study, the expression level of TGF-β1 was found to be correlated with Emax, Emean, Esd, Eratio, and the expression level of TGF-β1 in breast lesions with “stiff rim sign” was significantly higher than those without “stiff rim sign”. According to previous studies, TGF-β1 could promote the activation and production of CAFs [24, 25]. CAFs mainly synthesize and secrete ECM proteins and proteins related to ECM remodeling, which in turn promotes the excessive accumulation of ECM components and the remodeling of ECM structure [17, 25, 26]. TGF-β1 could also directly stimulate the synthesis and cross-linking rearrangement of collagen, elastin and laminin, and inhibit the activity of enzymes that degrade ECM components, leading to excessive accumulation of ECM components and structural changes of ECM [21, 27]. In addition, TGF-β1 could improve cell adhesion by promoting cancer cell synthesis and secretion of a variety of proteases, resulting in cancer cells adhering to the surrounding breast stroma and adipose tissue, and thus reduced lesion activity and increased lesion stiffness [28].

Our study also found that the expression levels of Smad2/3, Erk1/2, p38MAPK, JNK2, PI3K and AKT in breast lesions were correlated with that of TGF-β1, which further suggested that TGF-β1 might indeed participate in almost the whole process of occurrence, development, invasion and metastasis of breast lesions through signal transduction pathways such as TGF-β1/Smad, TGF-β1/MAPK, PI3K/AKT.

One of the important factors affecting the prognosis and 5-year survival rate of patients with malignant breast lesions was whether they had lymph node metastasis, especially axillary lymph node metastasis [29]. Our study showed that the expression levels of TGF-β1 and its signal molecules in malignant breast lesions had a certain value in predicting axillary lymph node metastasis, which is of great significance for axillary lymph node dissection in the clinical operation of malignant breast lesions.

It was also found in the study that the expression level of TGF-β1 was the main factor affecting the elasticity characteristics of breast lesions, and the expression level of TGF-β1 was the independent risk factor for the presence of “stiff rim sign”, which suggested that TGF-β1 might play an important role in the regulation of elasticity characteristics of breast lesions. TGF-β1 might become a breakthrough point for differential diagnosis of benign and malignant breast lesions and a potential target for treatment.

There are some limitations in this study. Firstly, the main components of ECM such as collagen fibers and elasticity fibers were not detected, so the correlation of collagen fibers, elasticity fibers, the expression levels of TGF- β1, signal molecules and the elasticity characteristics of breast lesions were not analyzed. Secondly, only the expression levels of signal molecules were stained, which indicates neither the activity of these factors nor activation of these factors by TGF-β1. Future studies need to focus on using antibodies against phosphorylated residues of such signaling molecules in order to indicate the activation of these factors. Thirdly, the main purpose of this study is to investigate the correlation of all the above-mentioned factors, the combined diagnostic performance research was not conducted. Therefore, the next step is to carry out the above three aspects of research, in order to make the study more in-depth and comprehensive.

The expression levels of TGF-β1, signal molecules of breast lesions showed good diagnostic performance and were correlated with the elasticity parameters. The expression levels of signal molecules were correlated with that of TGF- β 1, which speculated that TGF- β 1 might play an important role in the regulation of breast lesion elasticity parameters and multiple signal molecule expressions.

## Supplementary Information

**Additional file 1.**

**Additional file 2.**

**Additional file 3.**

**Additional file 4.**

## Data Availability

The datasets used and/or analyzed during the current study are available from the corresponding author on reasonable request.

## Abbreviations

SWE: Shear wave elastography
ECM: Extracellular matrix
TGF β: Transforming growth factor β
VAB: vacuum-assisted biopsy
CNB: Core needle biopsy
ROI: Region of interest
IOD: Integrated optical density
ROC: Receiver operator characteristic curve
AUC: Area under the curve
CAFs: Cancer associated fibroblasts
EMT: Epithelial-mesenchymal transformation
