# Supplementary material for: TGF-β1 and its signal molecules: are they correlated with the elasticity characteristics of breast lesions?
Source: BMC Cancer. 2021 Dec 15;21:1336. doi: 10.1186/s12885-021-09036-4 (PMC8675468; doi:10.1186/s12885-021-09036-4)
Supplement: Supplementary file 1 — Additional file 1. [file 12885_2021_9036_MOESM1_ESM.docx]

**Supplementary material 1**

**Expression level of TGF-β1 and elasticity values for different benign histological subtypes**

| Factors | Benign histological subtypes | | | | |
| --- | --- | --- | --- | --- | --- |
|  | **Fibroadenoma (*n*=32)** | **adenosis**  **(*n*=40)** | **intraductal papilloma**  **(*n*=3)** | **inflammatory lesions (*n*=8)** | **benign phyllodes tumor (*n*=1)** |
| Emax (kPa) | 57.7±36.7 | 56.1±54.2 | 47.8/36.4/44.2 | 60.3±55.5 | 161.9 |
| Emean (kPa) | 34.3±22.8 | 35.9±27.5 | 24.1/20.1/27.1 | 38.4±35.1 | 98.2 |
| Emin (kPa) | 13.4±8.4 | 17.1±16.7 | 12.7/16.7/17.4 | 15.2±12.8 | 10.2 |
| Esd (kPa) | 9.3±5.2 | 8.6±10.0 | 5.5/2.9/4.3 | 10.1±6.3 | 25.2 |
| Eratio | 2.0±1.0 | 1.8±1.2 | 2.8/1.8/13.3 | 2.2±1.2 | 6.2 |
| Stiff rim sign [n(%)] | 0 (0) | 1 (2.5) | 0 (0) | 2 (25) | 1(100) |
| TGF--β1 | 0.1012±0.0700 | 0.0964±0.0856 | 0.1012/0.0728/0.0675 | 0.1154±0.1007 | 0.2998 |

**Expression level of TGF-β1 and elasticity values for different malignant histological subtypes**

| Factors | Malignant histological subtypes | | |
| --- | --- | --- | --- |
|  | **Invasive carcinoma (*n*=46)** | **Intraductal carcinoma (*n*=3)** | **Mucinous carcinomas (*n*=2)** |
| Emax (kPa) | 155.9±86.1 | 159/216.6/216.1 | 168.6/105.9 |
| Emean (kPa) | 97.0±56.1 | 87.8/113.3/125.2 | 95.0/50.8 |
| Emin (kPa) | 18.2±20.9 | 54.3/41.8/34.3 | 12.7/4.3 |
| Esd (kPa) | 30.1±17.8 | 20.8/39.6/39.1 | 28.1/21.4 |
| Eratio | 6.2±4.5 | 4.0/4.5/5.2 | 9.3/1.3 |
| Stiff rim sign [n(%)] | 36 (78.3) | 1 (33.3) | 1 (50) |
| TGF--β1 | 0.2910±0.0840 | 0.3019/0.3486/0.3691 | 0.3138/0.2651 |
