# Supplementary material for: TGF-β1 and its signal molecules: are they correlated with the elasticity characteristics of breast lesions?
Source: BMC Cancer. 2021 Dec 15;21:1336. doi: 10.1186/s12885-021-09036-4 (PMC8675468; doi:10.1186/s12885-021-09036-4)
Supplement: Supplementary file 2 — Additional file 2. [file 12885_2021_9036_MOESM2_ESM.docx]

**Supplementary material 2**

**Efficacy of TGF-β1, Smad2/3, Erk1/2, p38 MAPK, JNK2, PI3K, AKT expression levels in the differential diagnosis of benign and malignant breast lesions**

| **Expression level** | **Cutoff Value** | **Sensitivity (%)** | **Specificity (%)** | **AUC** |
| --- | --- | --- | --- | --- |
| TGF-β1 | ＞0.2175 | 90.0 | 88.2 | 0.931 (0.874-0.967) |
| Smad2/3 | ＞0.1496 | 93.9 | 90.2 | 0.953 (0.901-0.982) |
| Erk1/2 | ＞0.2036 | 80.0 | 89.4 | 0.920 (0.861-0.960) |
| p38 MAPK | ＞0.1447 | 89.1 | 95.5 | 0.957 (0.902-0.986) |
| JNK2 | ＞0.1583 | 90.2 | 85.0 | 0.934 (0.874-0.971) |
| PI3K | ＞0.2624 | 93.6 | 94.4 | 0.960 (0.901-0.989) |
| AKT | ＞0.1230 | 95.7 | 84.1 | 0.939 (0.883-0.974) |
