# Supplementary material for: TGF-β1 and its signal molecules: are they correlated with the elasticity characteristics of breast lesions?
Source: BMC Cancer. 2021 Dec 15;21:1336. doi: 10.1186/s12885-021-09036-4 (PMC8675468; doi:10.1186/s12885-021-09036-4)
Supplement: Supplementary file 3 — Additional file 3. [file 12885_2021_9036_MOESM3_ESM.docx]

Supplementary material 4


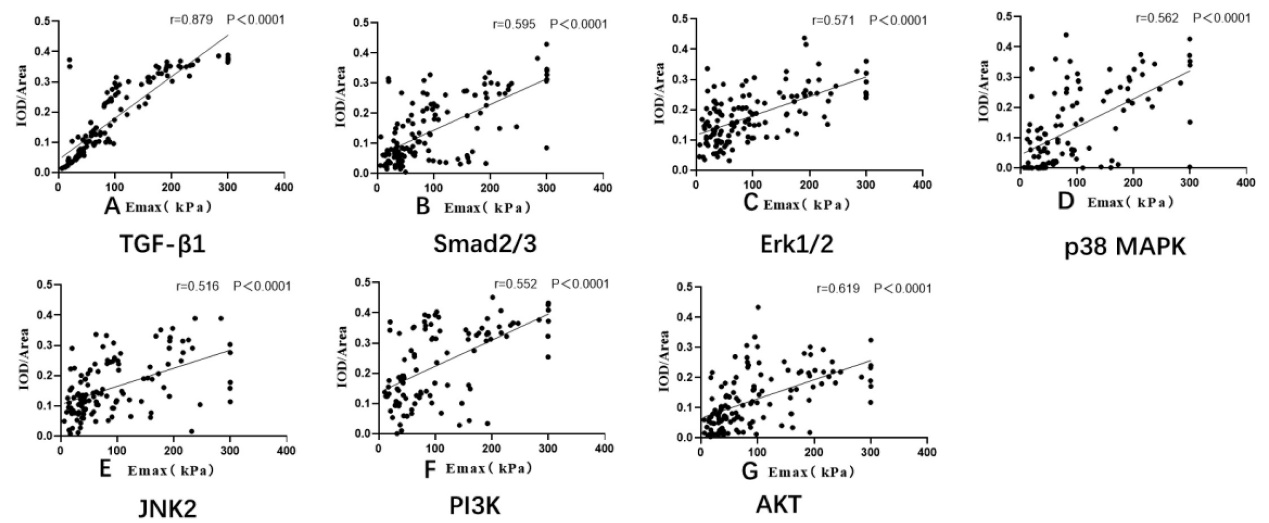
**Figure S1** Correlations of TGF-β1, Smad2/3, Erk1/2, p38 MAPK, JNK2, PI3K and AKT expression with Emax.


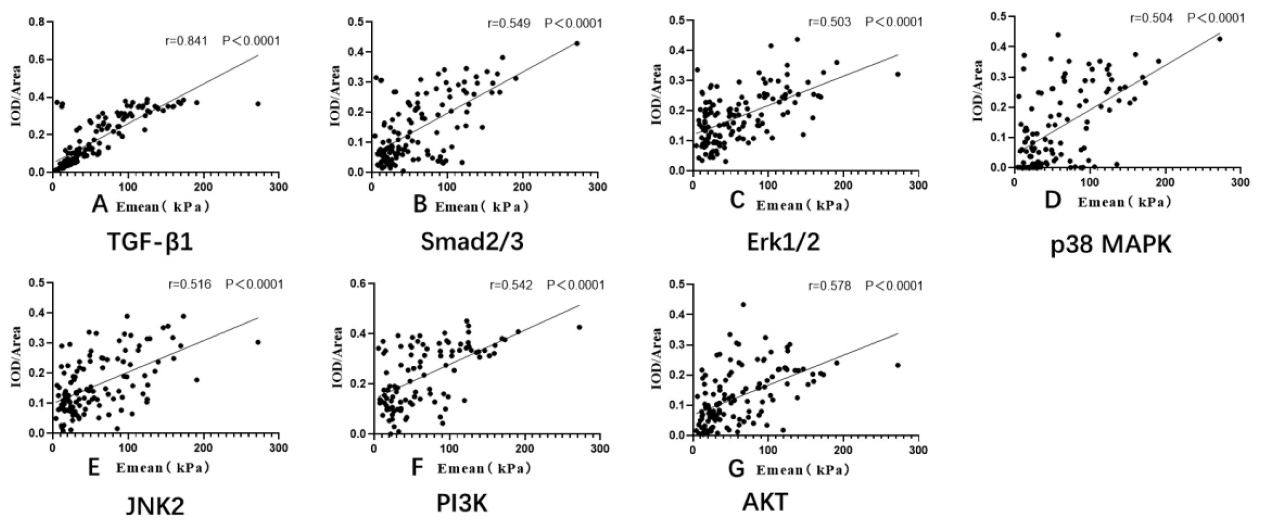
**Figure S2** Correlations of TGF-β1, Smad2/3, Erk1/2, p38 MAPK, JNK2, PI3K and AKT expression with Emean.


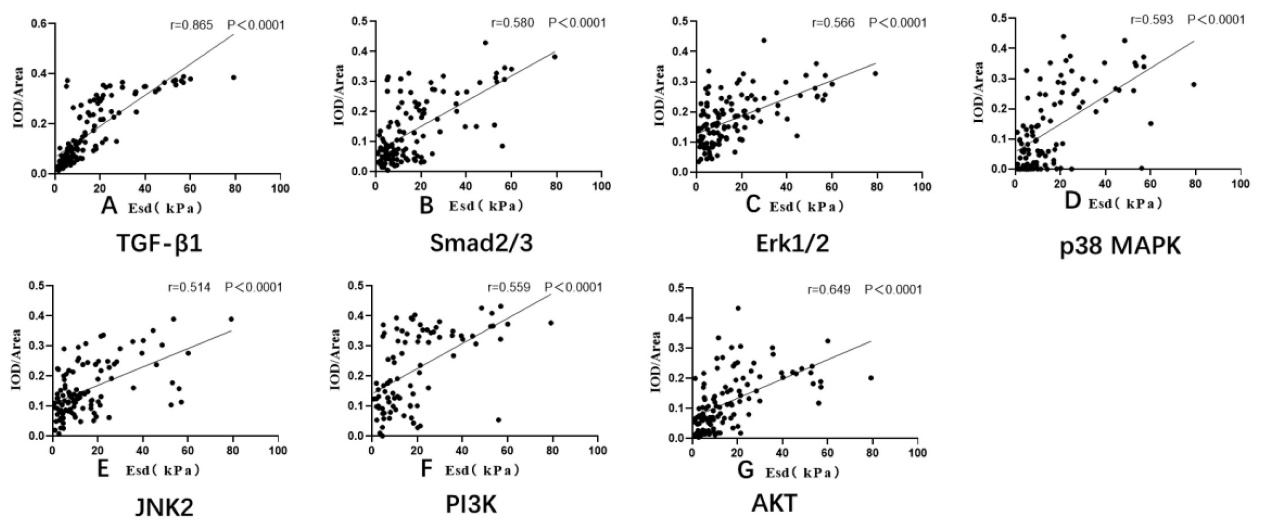
**Figure S3** Correlations of TGF-β1, Smad2/3, Erk1/2, p38 MAPK, JNK2, PI3K and AKT expression with Esd.


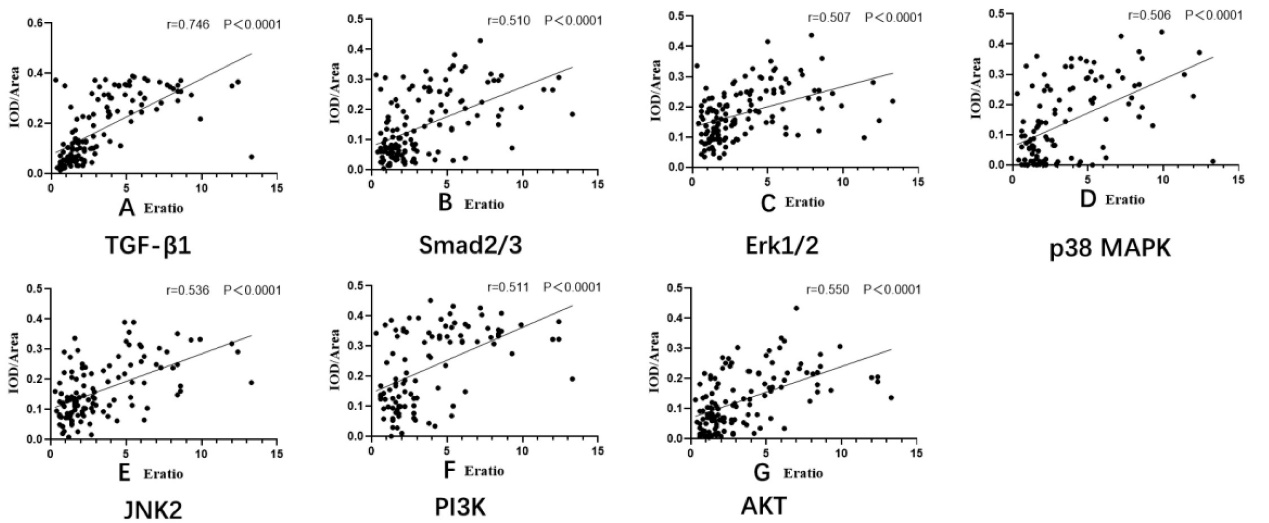
**Figure S4** Correlations of TGF-β1, Smad2/3, Erk1/2, p38 MAPK, JNK2, PI3K and AKT expression with Eratio.


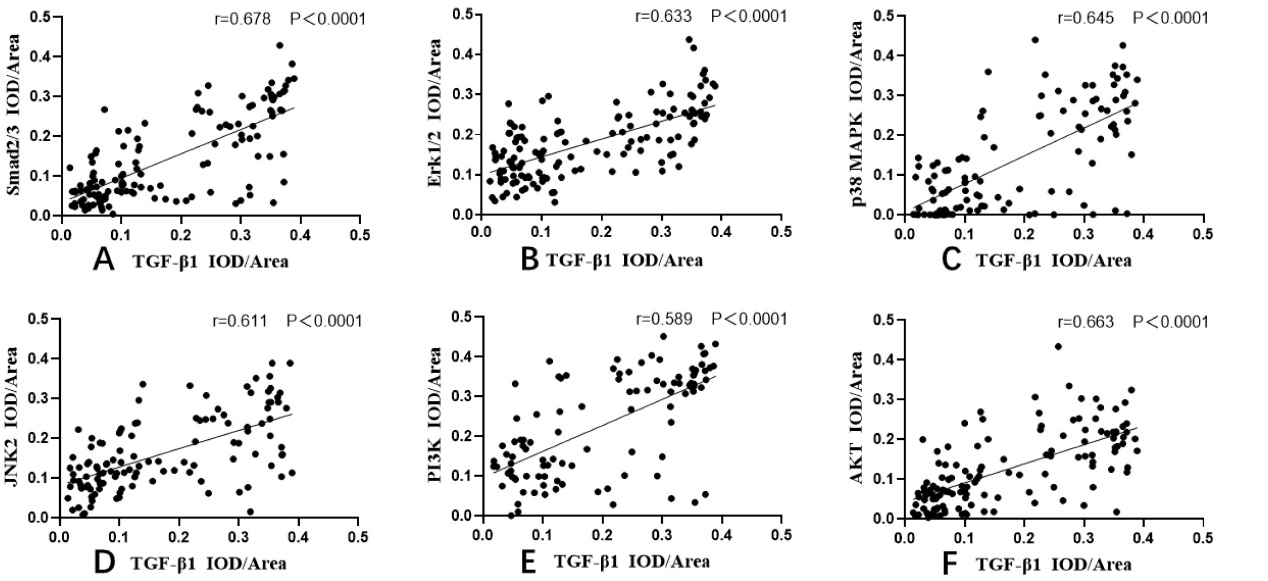
**Figure S5** Correlations of Smad2/3, Erk1/2, p38 MAPK, JNK2, PI3K and AKT expression with TGF-β1.
