# Supplementary material for: TGF-β1 and its signal molecules: are they correlated with the elasticity characteristics of breast lesions?
Source: BMC Cancer. 2021 Dec 15;21:1336. doi: 10.1186/s12885-021-09036-4 (PMC8675468; doi:10.1186/s12885-021-09036-4)
Supplement: Supplementary file 4 — Additional file 4. [file 12885_2021_9036_MOESM4_ESM.docx]

Supplementary material 3

**Efficacy of TGF-β1, Smad2/3, Erk1/2, p38 MAPK, JNK2, PI3K, AKT expression levels in the differential diagnosis of malignant breast lesions with or without axillary lymph node metastasis**

| **Expression level** | **Cutoff Value** | **Sensitivity (%)** | **Specificity (%)** | **AUC** |
| --- | --- | --- | --- | --- |
| TGF-β1 | ＞0.3138 | 86.7 | 83.3 | 0.853 (0.703-0.946) |
| Smad2/3 | ＞0.2679 | 80.0 | 62.5 | 0.697 (0.529-0.834) |
| Erk1/2 | ＞0.2457 | 80.0 | 58.3 | 0.694 (0.527-0.832) |
| p38 MAPK | ＞0.2812 | 69.2 | 65.2 | 0.706 (0.531-0.845) |
| JNK2 | ＞0.2760 | 50.0 | 80.0 | 0.654 (0.466-0.813) |
| PI3K | ＞0.3075 | 86.7 | 40.9 | 0.667 (0.493-0.813) |
| AKT | ＞0.2348 | 42.9 | 91.3 | 0.689 (0.516-0.831) |
